# *Candida albicans* necrotizing fasciitis following cosmetic tourism: A case report

**DOI:** 10.1016/j.jpra.2023.10.004

**Published:** 2023-10-05

**Authors:** Roberta Gilardi, Paola Parisi, Luca Galassi, Guido Firmani, Massimo Del Bene

**Affiliations:** a Division of Plastic & Reconstructive Surgery, San Gerardo Hospital of Monza, Italy; b Department of Plastic and Regenerative Surgery, San Gallicano Dermatological Institute IRCCS Rome, Italy; c Division of Vascular Surgery, San Gerardo Hospital of Monza, Italy; d Faculty of Medicine and Psychology, Sapienza University of Rome – Department of Plastic Surgery Sant'Andrea Hospital, Rome, Italy

**Keywords:** Medical tourism, Cosmetic tourism, Infective complications, Necrotizing fasciitis, Latissimus dorsi, Breast reconstruction

## Abstract

**Background:**

Necrotizing fasciitis is a rare and potentially life-threatening soft tissue infection, even more so when associated with fungal causative agents. Onset has been identified in nosocomial settings following elective surgery, but not in esthetic surgery.

**Case presentation:**

We here present a case of necrotizing fasciitis related to *Candida albicans* infection which occurred in an immunocompetent patient who received a cosmetic breast augmentation mastopexy combined with a Brazilian Butt Lift using autologous fat grafting. The case was managed with aggressive wound exploration and debridement. Treatment was delayed by the diagnostic challenge and the difficulty in identifying the causative agent, but the patient fully healed and recovered once the *C. albicans* was isolated in culture tests and appropriate antimycotic treatment was implemented.

**Conclusion:**

Considerations should be made regarding the possibility of implementing an antimycotic option for first-line empirical treatment despite the rarity of fungal etiology because of the threat of diagnostic delay and worse outcome.

## Introduction

Necrotizing fasciitis (NF) is a rare soft tissue infection, characterized by rapid but subtle onset of spreading inflammation and necrosis starting from the fascia and subcutaneous fat, with subsequent necrosis of the overlying skin and involvement of deep muscle layer if not promptly treated.^1^ Common presenting signs include fever, erythema, necrosis, seropurulent discharge, and ultimately sepsis. Three different types of NF have been described: type 1 is a polymicrobial infection due to at least one anaerobic and/or facultative anaerobes (Enterobacteriaceae, nongroup A Streptococci); type 2 is monomicrobial: group A β-hemolytic Streptococci and/or other Streptococci and/or Staphylococci; type 3 is related to marine Vibrio infection.^2^ NF secondary to *Candida spp.* infection is exceedingly rare with only few case reports published to date.^3^ Patients at increased risk of fungal NF are those who are immunocompromised, with poorly controlled diabetes, or who have peripheral vascular disease. We present a case of NF sepsis related to *C. albicans* infection which occurred in an immunocompetent patient who received cosmetic surgeries abroad.

## Case report

This case report adheres to the Strengthening The Reporting of OBservational studies in Epidemiology (STROBE) checklist. A 23 year-old woman with no previous medical history presented to the emergency department (ER) of our institution with breast pain and fever 7 days after cosmetic surgical procedures she received abroad. She underwent breast augmentation with mastopexy and a Brazilian Butt Lift in Turkey.

**Figure 1:**
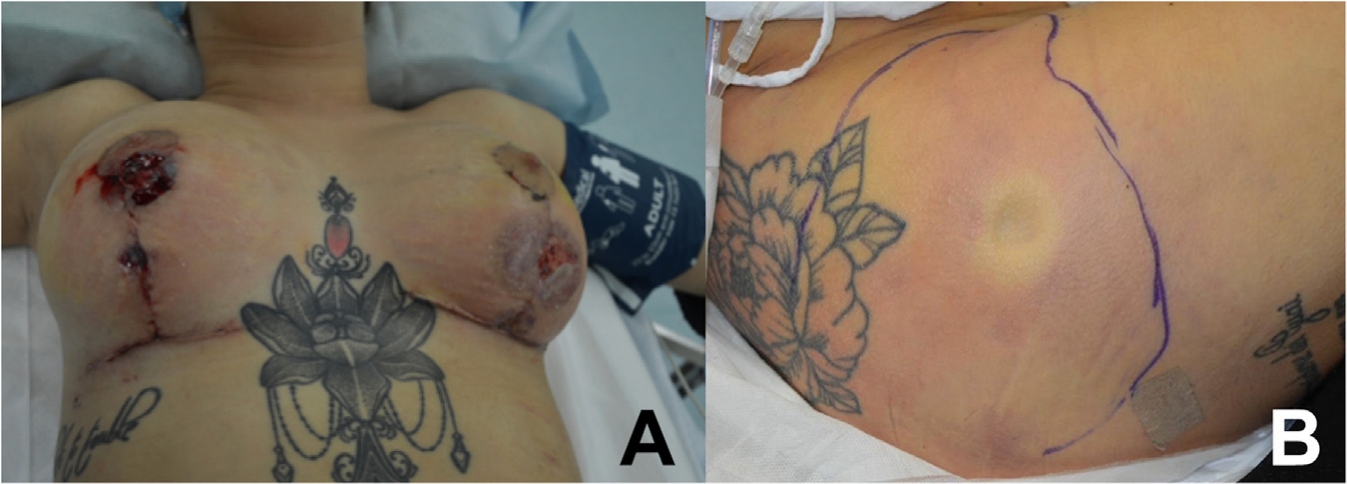
Clinical presentation of the patient to the emergency setting with necrotizing fasciitis affecting the breast (A) and the gluteal region (B), respectively after a breast augmentation with mastopexy and gluteal fat grafting from medical tourism.

No clinical documentation regarding the performed surgeries was available. However, the patient reported that she was discharged on post-operative day (POD) 1 with a temperature of 37.2 °C and antibiotic therapy consisting in Levofloxacin 500 mg once a day. She returned to Italy the next day, and 24 h later (POD 3) she noticed new onset of erythema around the vertical breast scars. On POD 7, she presented to the ER with widespread swelling of breast, mild erythema of the buttocks, and signs of general infection: blood pressure of 90/40 mmHg, heart rate of 140 bpm, O_2_ blood saturation of 96 % and body temperature of 40 °C. Blood tests showed marked leukocytosis and signs of systemic inflammation (white blood cells [WBC] = 15,000 × 10^9^/dL; C-reactive protein [CRP] = 30 mg/L) (Figure 1).

A surgical site infection was presumed so she was started on broad-spectrum intravenous antibiotic therapy consisting of Piperacillin-Tazobactam 4.5 g 4 times a day and Vancomycin 2 g once a day. The patient then underwent urgent bilateral breast prosthesis explantation. During surgery, an extensive liponecrotic pseudocyst and a purulent collection of the anterior thoracic wall were found and drained. Swabs and necrotic material were sent for testing, but no pathogens were isolated from samples obtained during surgery.

The next day, clinical conditions worsened with onset of respiratory and hepatic failure [WBC = 20.960 × 10^9^/dL; CRP 29 mg/mL; pO_2_ = 38 mmHg; pCO_2_ = 27.5 mmHg; pH = 7,51; Aspartate transaminase = 465 IU/L; Alanine transaminase = 383 IU/L], for which a transfer to the intensive care unit (ICU) was decided. The gluteal regions and thighs appeared red and swollen, with a progression of skin discoloration in the breasts where clear margins had been achieved during the first operation. A scan of the chest revealed pleural effusion that impaired ventilation. Due to the rapidly worsening clinical condition, a NF was presumed and antibiotic therapy was refined by replacing the previous regimen with Daptomycin 700 mg once a day, Meropenem 2 g 3 times a day and Clindamycin 600 mg 4 times a day, and a second surgical procedure was performed consisting in extensive debridement and fasciotomy which exposed necrotic tissues extending to the pectoralis major muscles and the lumbar region. All cavities were thoroughly washed with 10 L of saline solution using a pulsated lavage system. Surgical accesses of the breast and gluteal regions were managed with Vacuum Assisted Therapy with continuous irrigation (VAC instill). Swabs and necrotic material tested negative once again.

**Figure 2:**
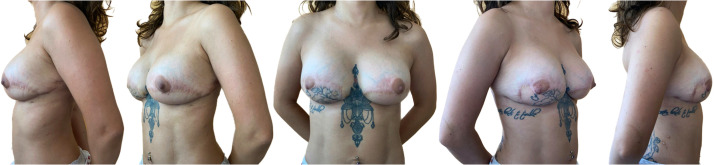
Breast reconstruction following aggressive wound debridement of the breast, using autologous bilateral latissimus dorsi flap, followed 6 months later by delayed bilateral breast implant placement for volume enhancement, in the frontal, oblique and side view.

The next 3 days were followed by multiorgan dysfunction syndrome onset, with renal failure and further enlargement of the necrotic breast areas which warranted a third surgical debridement. The intraoperative cultural samples this time tested positive for *C. albicans* which was also isolated in urine cultures and bronchoalveolar aspirate. Thus, a systemic antifungal therapy with Caspofungin IV injections was started at 70 mg once a day. During the following 2 weeks, clinical conditions progressively improved, with partial growth of granulation tissue on both the thigh and breast wounds, which were treated with collagenase-based dressings.^4^ The gluteal region was then closed with direct sutures. One month after her admission, the patient received bilateral breast reconstruction using the latissimus dorsi myocutaneous flap. At POD 45 from her initial surgeries, the patient was discharged after full recovery. She returned 6 months later and received a delayed placement of smooth round mentor (Johnson & Johnson, New Brunswick, New Jersey, USA) silicone breast implants with a moderate plus profile and a volume of 250 cc to enhance her breast mound, with an uneventful post-operative course and satisfactory result (Figure 2).

## Discussion

Skin breach from surgical intervention has been identified as a possible point of entry for the development of NF, making nosocomial cases a potential sequelae of elective surgery.^5^ This includes even esthetic surgery, although this occurrence is exceptionally rare since cosmetic surgery patients are generally in good health. This explains why it has only been occasionally reported in literature to date. In fact, only two cases of NF have been described after breast augmentation, with one of them having also received an abdominal liposuction.^6^^,^^7^

The case we described is noteworthy for the fact that it combines an already rare occurrence, being a NF infection following cosmetic surgery, with an even rarer one, which is NF associated with fungal infections.^8^ Invasive fungal NF is a life-threatening surgical emergency associated with high morbidity and mortality. It is characterized by atypical clinical presentations, and has been linked with immunocompromised states caused by either acquired conditions such as uncontrolled diabetes,^9^ or congenital disorders, namely severe combined immunodeficiencies (SCID).^10^ The severity of fungal NF is due to either the diagnostic challenge which delays recognition and treatment, antimycotic resistance of the isolated species, or involvement of certain anatomical locations such as the head and neck.

## Conclusions

This case taught us that although extremely rare, fungal NF following a cosmetic surgery procedure in an immunocompetent patient is possible. Despite the fulminant onset, aggressive surgical exploration with wound debridement coupled with antimycotic treatment were successfully used to manage the infection. The maiming sequelae from the loss of substance in the breast areas were managed with a bilateral latissimus dorsi-based reconstructive procedure which allowed wound closure and recovery. Considerations should be made regarding the possibility of implementing an antimycotic option for first-line empirical treatment despite the rarity of fungal etiology because of the threat of diagnostic delay and worse outcome.

## Statement of human and animal rights, or ethical approval

This article does not contain any studies with human participants or animals performed by any of the authors.

## Informed consent

The patient whose case is presented has provided her informed consent for the publication of the manuscript.

## Declaration of Competing Interest

The authors declare that they have no conflicts of interest to disclose.

## Appendix Supplementary materials

Image, image 1

Image, image 2
