# Supplementary figures and images for: Candida albicans necrotizing fasciitis following cosmetic tourism: A case report
Source: JPRAS Open. 2023 Oct 5;38:129–33. doi: 10.1016/j.jpra.2023.10.004 (PMC10587449; doi:10.1016/j.jpra.2023.10.004)

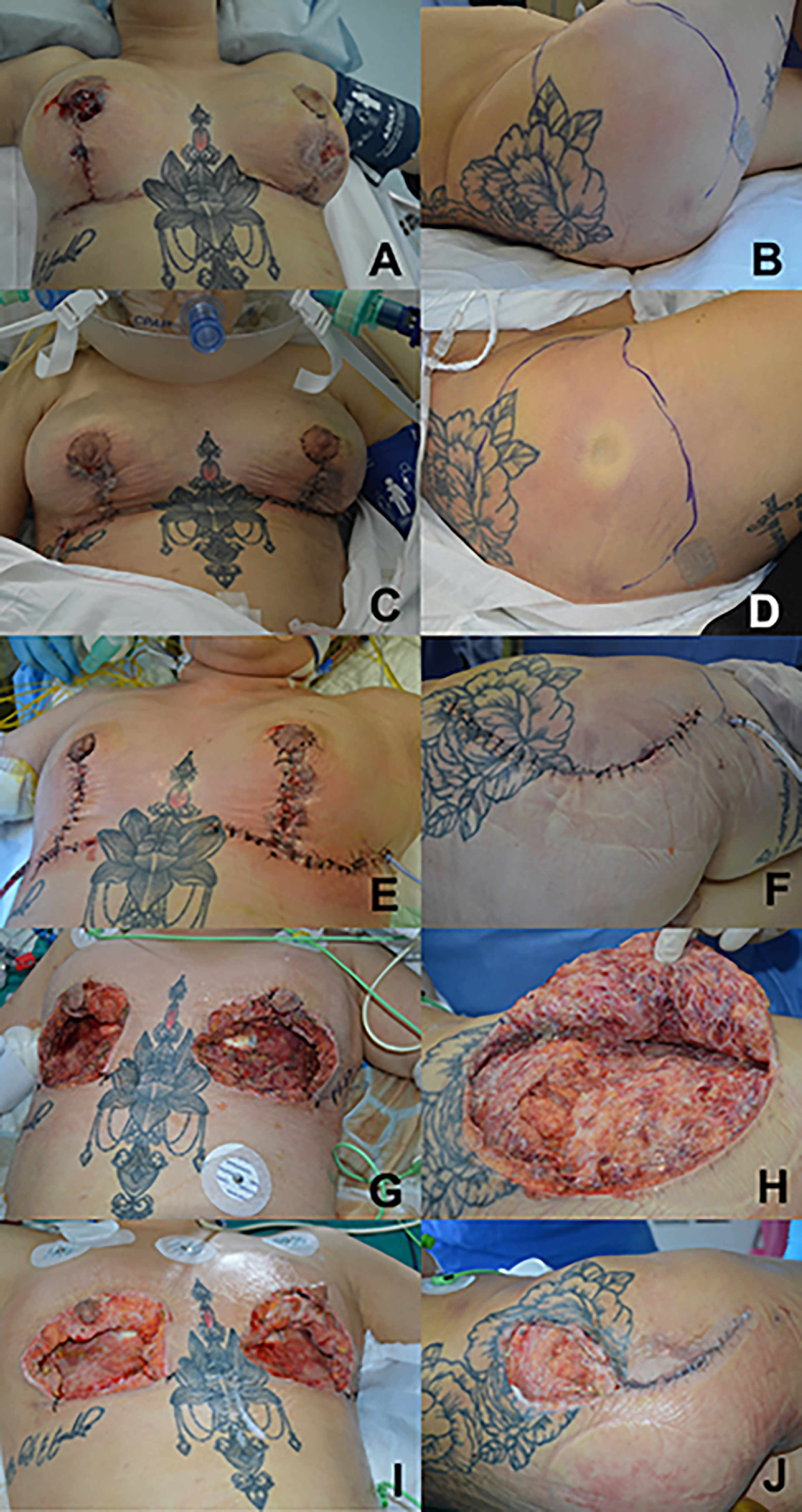

Supplement: Supplementary file 1 [file mmc1.jpg]

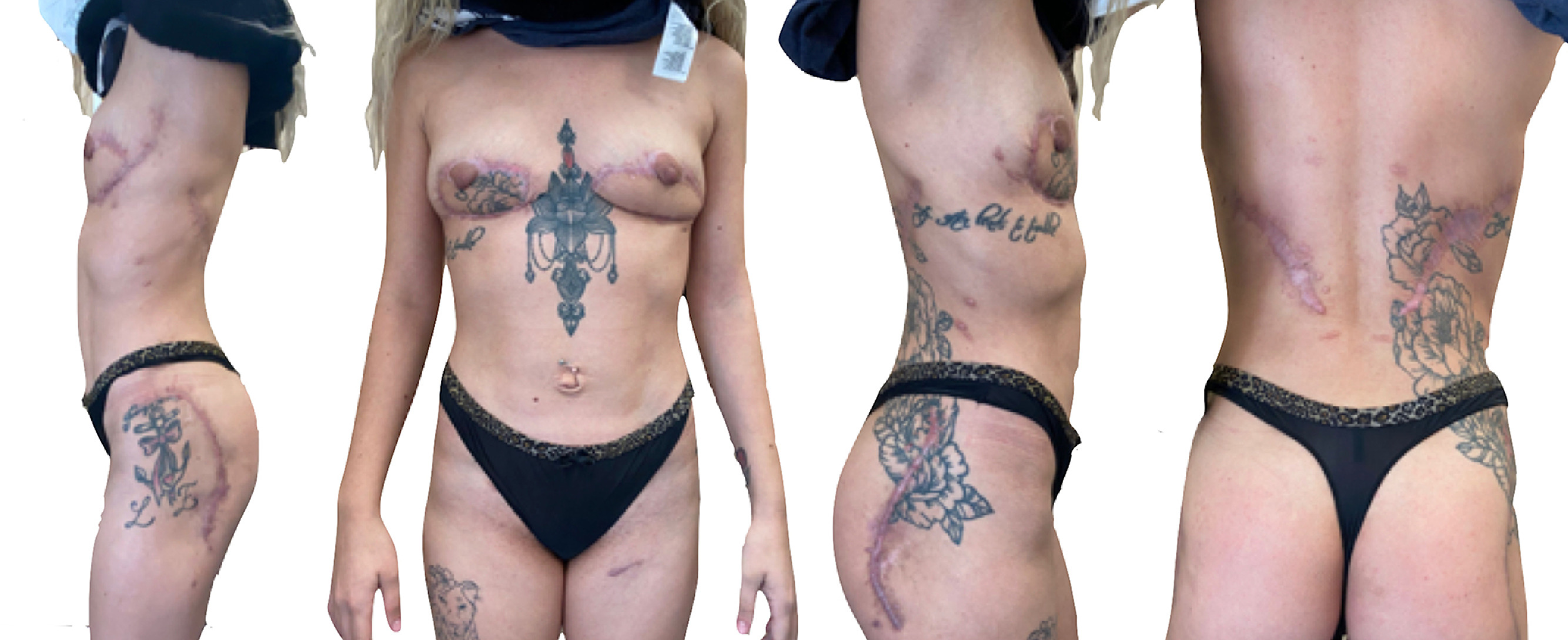

Supplement: Supplementary file 2 [file mmc2.jpg]
